# *Semimytilus algosus*: first known hermaphroditic mussel with doubly uniparental inheritance of mitochondrial DNA

**DOI:** 10.1038/s41598-020-67976-6

**Published:** 2020-07-09

**Authors:** Marek Lubośny, Aleksandra Przyłucka, Beata Śmietanka, Artur Burzyński

**Affiliations:** Department of Genetics and Marine Biotechnology, Institute of Oceanology Polish Academy of Sciences, Sopot, Poland

**Keywords:** Next-generation sequencing, Mitochondrial genome, Mitochondria, Phylogenetics

## Abstract

Doubly uniparental inheritance (DUI) of mitochondrial DNA is a rare phenomenon occurring in some freshwater and marine bivalves and is usually characterized by the mitochondrial heteroplasmy of male individuals. Previous research on freshwater Unionida mussels showed that hermaphroditic species do not have DUI even if their closest gonochoristic counterparts do. No records showing DUI in a hermaphrodite have ever been reported. Here we show for the first time that the hermaphroditic mussel *Semimytilus algosus* (Mytilida), very likely has DUI, based on the complete sequences of both mitochondrial DNAs and the distribution of mtDNA types between male and female gonads. The two mitogenomes show considerable divergence (34.7%). The presumably paternal M type mitogenome dominated the male gonads of most studied mussels, while remaining at very low or undetectable levels in the female gonads of the same individuals. If indeed DUI can function in the context of simultaneous hermaphroditism, a change of paradigm regarding its involvement in sex determination is needed. It is apparently associated with gonadal differentiation rather than with sex determination in bivalves.

## Introduction

Bivalvia is a class of hinge-shell animals, that are broadly used in industry as a food source, pearl producers or diet supplements^1^, and have high ecological value as water quality bioindicators^2^ and sequesterers of carbon dioxide^3^. They are interesting also in terms of molecular biology. Their byssus proteins^4^ (thread-like adhesive structures attaching those animals to the rocky sea bottom) could potentially be used in industry and medicine^5,6^. They are one of the few groups of animals after Tasmanian devils^7^, dogs^8^ and Syrian hamsters^9^ where transmissible cancer was observed^10,11^. Furthermore, many species, more than 100 known so far^12^, show doubly uniparental inheritance of mitochondrial DNA (DUI)^13–15^, system uniquely different from strict maternal, paternal or biparental inheritance. In DUI two highly divergent, from 10% to over 50%^16^, mitochondrial DNA types are present in male individuals. Females are homoplasmic (F-type mtDNA) and males are heteroplasmic (F-type present in somatic tissues and M-type dominating gonads). Progeny receive different types of mitochondria through homoplasmic gametes: F from eggs and M from sperm^17^. Depending on the progeny’s sex the mitochondria from the father are either lost (female progeny), or are grouped together, becoming the dominant fraction in the male gonad^18^ (male progeny).

With the expanding knowledge on taxonomic distribution of this phenomenon, evolutionary studies try to answer the question of the DUI origin: how many times DUI evolved, whether it is a bivalvian synapomorphy or whether it was lost and re-invented several times independently^14,19,20^. Studies concerning molecular mechanisms involve quests for candidate DUI-related genes in transcriptomes^21,22^ primarily focusing on mtDNA-derived sequences, often featuring additional mitochondrial open reading frames (ORF) and extensions of mitochondrial genes^21,23–27^. Studies have shown that these ORFs are indeed transcribed and translated, implying their role in the segregation of mitochondria^27–29^. It has also been suggested that DUI evolved primarily as a sex determination system^30^, with the mitogenome influencing the sex of the progeny. Hermaphroditic species of freshwater mussels (Unionoida), lose not only the paternally inherited M mitogenome but also the gender-specific open reading frame in the remaining F type mitogenome. Following this, the hermaphroditic freshwater mussel *Anodonta cygnea* does not have supranumerary open reading frames in the mitogenome^31^.

*Semimytilus algosus* (Gould, 1850) is a small, marine mussel living on wave-exposed rocky shores along the Pacific coast of South America as well as on the western (Atlantic) coast of Africa, where it has been marked as an invasive species^32,33^. The species is a simultaneous hermaphrodite with male and female gonads located in the mantle tissues on the opposite sides of the body (different shell halves): the grey-violet mantle contains female gonads and the whitish mantle contains male gonads^34^. As we show in the following article it is the first known hermaphroditic mussel possessing doubly uniparental inheritance, which indicates that DUI is connected with gamete differentiation rather than with sex determination in bivalves.

## Results

### mtDNA

**Figure 1:**
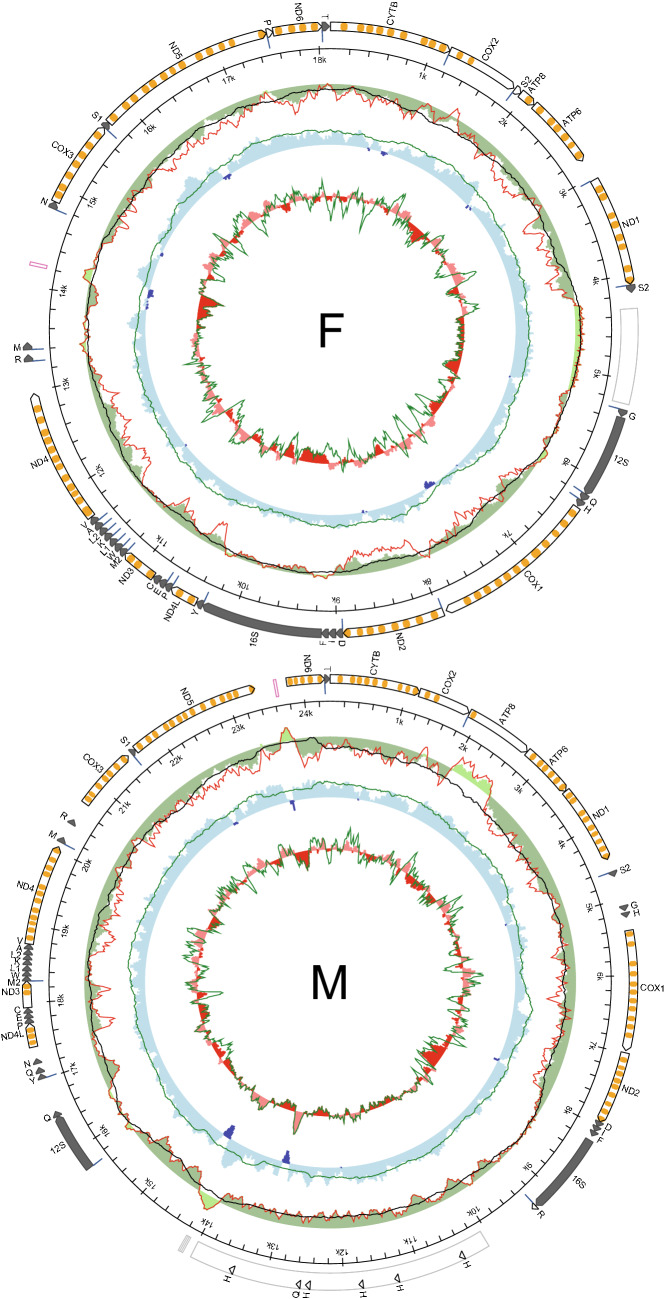
Genetic map of *Semimytilus algosus* mitochondrial genomes. The white arrows represent protein coding genes with predicted transmembrane domains of encoded proteins marked in orange. The long dark arrows represent rRNA genes; the short dark arrows represent tRNA labeled by a one letter amino acid code, short white arrows represent duplicated tRNA-like structures, a pink box indicates the location of an AT-rich region; a grey box indicates the location of repetitive sequences; blue lines in front of the genes show the location of the polyadenylation signal. Inner circles represent local compositional bias, calculated in a 200 bp long sliding window with 25 bp steps, unless indicated otherwise. A green outer circle represents a AT-skew (A−T/A+T); a red line represents a filtered AT‐skew, calculated at non-coding regions and the second codon position only. A black line represents a filtered AT-skew, calculated at neutral and non-coding positions only, in a larger, 1000 bp long window. A middle blue circle represents a GC skew (G−C/G+C), and a green line represents a GC skew at neutral sites, calculated in a window of 1,000 bp. Both skew indices are presented in absolute scale, starting at zero. An inner red circle represents local GC content, and a green line shows GC content at neutral sites. Local GC content is presented in scale relative to the average for the whole mitogenome.

Two divergent mitogenomes were detected in the male and female gonads of an individual *S. algosus* mussel. Both were sequenced and annotated. We will refer to the mitogenome dominating male gonads as M, and the other as F. The M and F mitogenomes (Fig. 1) differ in length. The F mitogenome is shorter (18,113 bp) than the M mitogenome (24,347 bp). Both mitogenomes encode all genes on a single strand: 13 protein-coding genes, 2 *rRNA* genes, and at least 23 *tRNA* genes were identified, including a second *tRNA* for methionine with TAT anticodon. Despite the length difference, the structure of the coding part is similar. One major difference concerns the small subunit *rRNA* gene. *12S rRNA* in the F genome is located between *nad1* and *cox1* genes whereas in the M mitogenome it is located in the region between the *16S rRNA* and *nad4l* genes. In addition to that, *atp8* in M mitogenome is exceptionally long (885 bp). There is no indication of polyadenylation inside its transcript, which would allow shorter annotation, although the transcript coverage (Table S1) in this region is quite low (8.82 ± 5.07). Minor structural differences between the M and F mitogenomes involve different relative locations of some *tRNA* genes: *tRNA*^*Asn*^, *tRNA*^*Arg*^ and *tRNA*^*Met*^. Finally, the non-coding regions have different length and localization in the two mitogenomes. In the M mitogenome a major non-coding region, very long and rich in repetitive sequences, is located between the two *rRNA* genes whereas in the F mitogenome the longest non-coding region is between *tRNA*^*Met*^ and *tRNA*^*Asn*^ and does not contain repetitive sequences. There are several other, shorter non-coding regions in both mitogenomes, none with any level of between-genome similarity.

In the F mitogenome two cases of possible duplication of *tRNA* genes were observed. The first case concerns the *tRNA*^*Pro*^-like structure located before *nad6.* This gene has a larger p-distance than true *tRNA*^*Pro*^ (0.377) but has conserved the anticodon loop structure. The second case concerns *tRNA*^*Ser2*^-like sequence located before *atp8* gene. This one has smaller p-distance than true *tRNA*^*Ser2*^ (0.147), but anticodon sequence is modified from AGA to AGC. In each of the two cases the polyadenylation signal marks beginning of the *tRNA*-like gene and there is no corresponding sequence in the M mitogenome.

There are several *tRNA*-like genes in the M mitogenome, too. They all involve *tRNA*^*Gln*^ or *tRNA*^*His*^ specificities. The two possible candidates for *tRNA*^*Gln*^ genes: one directly behind *12S rRNA* and the second between *tRNA*^*Tyr*^ and *tRNA*^*Asn*^, are difficult to differentiate, similar p-distance 0.344 and 0.388 and Gibbs free energy (dG − 9.4 and − 10.4 kcal/mol respectively) for clover leaf structure. Since there is no clear way to discriminate one over another both will be treated as possibly valid *tRNA* genes. Apart from the *tRNA*^*His*^ gene located before *cox1,* as in the F mitogenome, there are several *tRNA*^*His*^-like structures in repetitive non-coding region of the M mitogenome. Some of them show the most stable structure (lowest dG) but the one before *cox1* gene, the most similar to *tRNA*^*His*^ from the F mitogenome (p-distances of 0.571 and 0.339 respectively) has been annotated as the correct one.

We were able to identify the polyadenylation sites (pA) in the F mitogenome, indicating the span of the mature transcripts. All protein and *rRNA* genes produce individual transcripts, except for *atp8* and *atp6*, which apparently form a bicistronic transcript. Unfortunately, for the M mitogenome RNA-seq coverage was much lower and only few pA sites were detected: after *16S rRNA* before long repetitive region, after repetitive region before *12S rRNA* and at boundaries of some *tRNA* genes (Fig. 1).

### Divergence and phylogenetics

**Figure 2:**
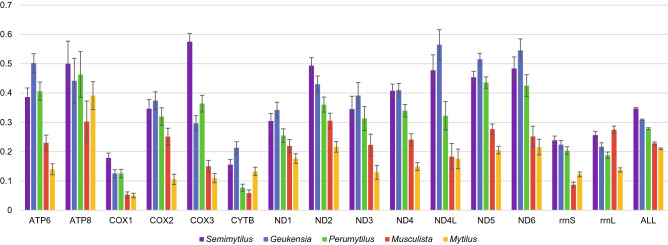
Average distance (p-distance) between protein and gene sequences from M and F genomes in Mytilidae. Violet bars represent *Semimytilus algosus*, blue bars represent *Geukensia demissa* (two genomes), green bars represent *Perumytilus purpuratus* (four genomes), red bars represent *Musculista senhousia* (two genomes), and yellow bars represent the *Mytilus edulis* complex (six genomes). Each gene sequence was extracted and aligned separately. The average between‐group nucleotide p‐distance was calculated in MEGA7. Standard error estimates were calculated by bootstrap procedure (10,000 replicates). For protein coding genes, amino acid p‐distances are shown; for RNA genes and the overall distance, the nucleotide p-distances are shown.

**Figure 3:**
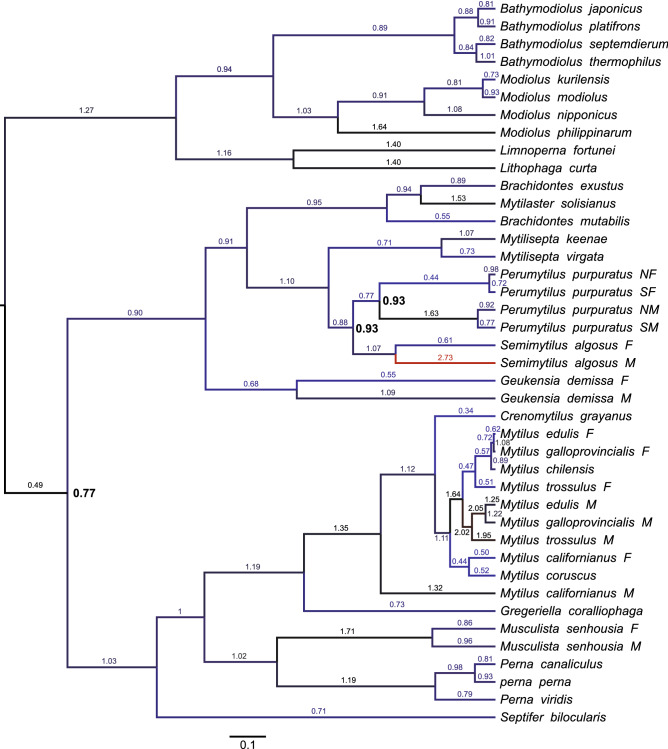
The phylogenetic position of *Semimytilus algosus* mitogenomes among mitogenomes of other mytilid mussels. Posterior probabilities of all bipartitions were 1.0, except for the nodes indicated by the bolded number. Numbers on branches represent evolutionary substitution rate.

The overall divergence (p-distance) between F and M genomes (Fig. 2) is quite high (0.347) achieving an unusually high value for cox3 protein, almost two times higher than for the F and M genomes of *Perumytilus purpuratu*s. Beside this protein, *12S rRNA*, atp8 and nad2 pairs are slightly more divergent in *S. algosus* than their counterparts in other known DUI possessing mytilids. Nevertheless, in the phylogenetic reconstruction (Fig. 3) the separation of F and M lineages in *S. algosus* is more recent than in *Geueknsia demissa* or *Perumytilus purpuratus* even though their overall M to F divergence (p-distance) is lower then for *S. algosus*. This is because the evolutionary rate in the M lineage of *S. algosus* is substantially higher than that of other mytilids, indicated here by values on phylogenetic tree branches.

To ascertain the intrapopulation divergence and compare selective pressure acting on F and M mitogenomes, three fragments from each mitogenome from eighteen individuals were amplified and sequenced (Table 1). A higher level of polymorphism has been observed for the genes from the F mitogenome. The ratios of non-sysonymous and synonymous substitutions and polymorphisms suggest strong purifying selection acting on both mitogenomes.

**Table 1:** Standard indices of genetic diversity in the coding sequences of ND5 (1,317 bp), ND6 (417 bp) and CYTB (663 bp) region and in the concatenated set of ND5, ND6, CYTB sequences for *Semimytilus algosus* mussel.

mt genome	Region	N	h	S	hd	θ	π	Tajima's D	Ka	S.E	Ks	S.E	Ka/Ks
F	ND5	18	18	111	1.00	0.0247	0.0156	− 1.5538	0.0009	0.0004	0.0643	0.0069	0.0140
F	ND6	18	18	34	1.00	0.0251	0.0137	− 1.8459*	0.0018	0.0009	0.0529	0.0106	0.0340
F	CYTB	18	18	43	1.00	0.0202	0.0100	− 2.0707*	0.0002	0.0002	0.0414	0.0065	0.0048
F	ND5, ND6, CYTB (concatamer)	18	18	188	1.00	0.0235	0.0137	− 1.7692	0.0009	0.0002	0.0558	0.0047	0.0161
M	ND5	18	8	17	0.83	0.0038	0.0032	− 0.5852	0.0003	0.0003	0.0122	0.0035	0.0246
M	ND6	18	6	6	0.78	0.0042	0.0045	0.2480	0.0004	0.0003	0.0175	0.0083	0.0229
M	CYTB	18	5	10	0.71	0.0044	0.0059	1.2481	0.0000	0.0000	0.0242	0.0083	0.0000
M	ND5, ND6, CYTB (concatamer)	18	9	33	0.86	0.0040	0.0042	0.1626	0.0002	0.0002	0.0164	0.0033	0.0122

N—represent the number of individuals, h—the number of identified haplotypes, S—the number of segregating sites, hd—haplotype diversity, θ—number of segregation sites in sample of N DNA sequences, π—nucleotide diversity, Tajima's D—neutrality test, *—represent statistical significance for *p* < 0.05, Ka—nonsynonymous substitutions, Ks—synonymous substitutions.

### qPCR

**Figure 4:**
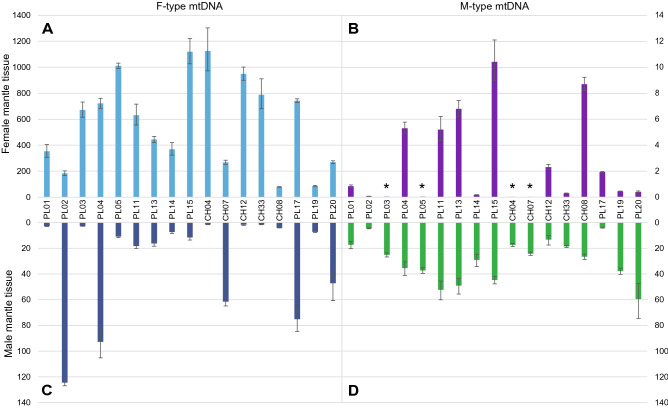
Number of mitochondrial genomes (F *cox1* and M *nad1*) in relation to copies of nuclear DNA (*atpα*). A. F mtDNA to nuclear DNA ratio in the Female mantle; B. M mtDNA to nuclear DNA ratio in the Female mantle; C. F mtDNA to nuclear DNA ratio in the Male mantle; D. M mtDNA to nuclear DNA ratio in theMale mantle. * asterisks show samples where the M mtDNA copy number approaches zero.

**Figure 5:**
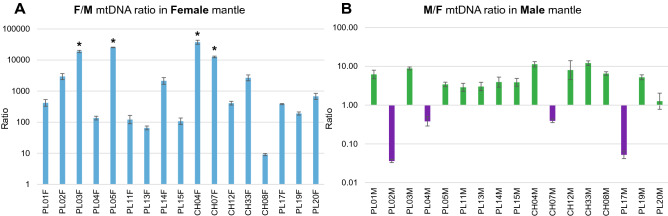
Ratios of M to F and F to M mitogenomes in tissues. A. F to M mtDNA ratio in Female mantle issues; B. M to F mtDNA ratio in Male mantle; * asterisks show samples where the M mtDNA copy number approaches zero, hence in those samples the F/M ratio lies between infinity and the marked value. All results were presented in logarithmic scale.

The distribution of the M and F mitogenomes in gonads/mantles (gonads and mantles are inseparably connected) was measured by qPCR with relation to a single-copy nuclear gene (Fig. 4). The M mitogenome was present in appreciable concentrations only in male gonads, although the exact value varied. The ratios of M to F concentrations were also calculated (Fig. 5). There is from hundreds, up to more than ten thousand times more F-type mtDNA than M-type mtDNA in female mantles, and, excluding the four cases, there was from three to twelve times more M-type mtDNA than F-type mtDNA in male mantle tissues. The four exceptional cases had more F-type mtDNA in the male mantle tissue, but only up to 30 times more. In the male gonad of individual PL02M, which apparently has the smallest M/F ratio in male mantle, it was estimated at 27.59 (CI: 25.03–30.42). The same tissue sample was used in next generation sequencing (NGS), and the ratio derived from sequencing the data was 31.05 (26.77–36.18), in good agreement with the qPCR estimate.

**Figure 6:**
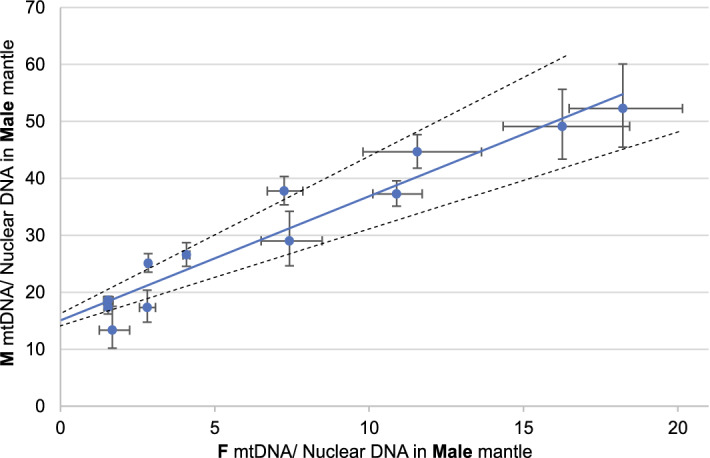
The correlation of the M and F mtDNA copy number in male tissues based on qPCR results. Intersection of the regression line with the Y axis marks predicted copy number of M mitogenomes in single sperm cell. Dotted line shows standard deviation.

Due to the lack of live specimens there was no possibility to check the precise mtDNA content in separated and purified sperm cells. In this species mantle tissues contain both somatic and generative cells. As a result, assuming that sperm cells are homoplasmic^15,35,36^ for M-type mtDNA, and that F-type mtDNA in mantle represent somatic cells' contribution, estimation of the mtDNA content in spermatozoa is possible. The plot of M-mtDNA/nDNA in the male mantle against F-mtDNA/nDNA in the same tissue (qPCR data) extrapolated to the zero should give an estimate of the M-mtDNA/nDNA ratio of pure sperm. The result is a straight trend line showing reasonably high correlation R^2^ = 0.9238 (Fig. 6) and crossing the Y axis at 15.06 (14.11- 16.26). It means that each sperm cell contains approximately 15 copies of M-mtDNA. Assuming that there are five mitochondria per sperm cell^18,37^, there should be around three M-mtDNA molecules in each sperm mitochondrion.

## Discussion

The two mitogenomes isolated from *S. algosus* both show a fairly typical structure. There were only minor difficulties with the certainty of some *tRNA* gene annotations, otherwise the complete set of metazoan mtDNA encoded genes was unambiguously identified in both. Based on the phylogenetic analysis (Fig. 3) the possibility that one of the mitogenomes comes from a contaminating, unrelated organism can safely be discarded: the two mitogenomes, despite the substantial genetic distance, form a sister relationship on the tree. Moreover, there are only a few structural differences between them, which could be explained by translocations involving primarily *12S rRNA* and three *tRNA* genes. A similar amount of structural differences between M and F mitogenomes is known in freshwater mussels^28^, but was not reported from a mytilid bivalve so far.

DUI is often associated with lineage-specific features present in the mitogenomes. With this respect, the presence of the very long *atp8* gene in M mitogenome is a possible candidate in *S. algosus*. This gene does vary in length in bivalves but in *S.algosus* it is extreme: over two times longer than in *Mytilus*^38–40^. Moreover, few 7–9 amino acid long repeats are present at the C-end of the hypothetical protein (Fig. S1). This gene extension could potentially be involved in mitochondria tagging mechanism, as postulated for similar extensions in other DUI bivalves^24,25,28,29,41–43^. Additional open reading frames (ORFs), as well as mitochondrial sequence coverage with RNA transcripts have been marked on supplementary Fig. 2 (Fig. S2). With gathered data it is hard to speculate about possible involvement of those ORFs in mitochondrial DNA inheritance.

All other genes have similar length. With one exception they also follow a similar pattern of divergence (Fig. 2). The exceptionally divergent cox3 (nearly 60% p-distance at amino acid level) is located close to an AT-rich region in both mitogenomes, and must have been involved in translocation events. These could explain the increased relative mutational pressure, but the observed genetic distance could also be a result of metabolic remodelling, recently shown to involve OXPHOS complexes IV and V in DUI bivalves^44^.

Reported amounts of mtDNA copies per cell^45–47^ vary greatly in different organisms, cell types and stages of oogenesis, from less than one thousand to hundreds of thousends^46,47^. For bivalves information about mtDNA copy number is scarce and indirect^36,48,49^, but the high concentrations of F-mtDNA in the female mantle observed in *S. algosus* are not surprising. The expectation from DUI model would be the exclusive presence of M mtDNA in sperm and ubiquitous presence of F mtDNA in other tissues^15,35,36^. We observed marked differences between individuals, particularly with respect to F mtDNA content in male mantle (Fig. 4). These may represent different stages of gametogenesis or different extents of contribution from the adjacent somatic tissues, in agreement with the model. The M-type mtDNA was consistently present in male mantle (29.2 ± 15.7 copies per nuclear genome). Very similar values were reported from male gonads of *Venerupis philippinarum* (32.25 ± 16.63)^36^. Even if the differences in the anatomy of these bivalves and the different choice of the reference gene make direct comparison of these results difficult, they do fulfill the expectation of the DUI model. The occasional presence of M-mtDNA in the female mantle is more problematic. It is commonly assumed that females and somatic tissues in DUI males are free from M-type mtDNA^50^, but depending on the individual species and molecular technique used, exceptions to this rule were also shown^36,51^. In the case of the hermaphroditic *S. algosus,* M-type mtDNA is always present in one of their mantles, so the contamination with small amounts of M-mtDNA is apparently difficult to avoid. The detected concentrations were always very low, consistent with this view.

To ascertain the possibility that the observed mitochondrial sequences represent *numts* rather than true mitogenomes, the polymorphisms of both mitogenomes were measured at a population level. At this level mitochondrial genes show a strong signature of purifying selection, evidenced by omega values much smaller than one^52^. For the two representative fragments of both *S. algosus* mitogenomes the observed polymorphism was also consistent with strong purifying selection (Table 1), supporting the view that these sequences are not *numts*. The overall level of polymorphism was very low, particularly in the M data set. The M mitogenome usually shows higher polymorphism than the F mitogenome^42,53^ especially for larger data sets^52,54^, but there is a known case of the mytilid *Musculista senhousia*^41^, where the situation is opposite. These anomalies can be explained by compensation draft feedback (CDF) process acting on the DUI tripartite genome, with more frequent selective sweeps in the M lineage periodically lowering its polymorphism^39^. Consequently, the evolutionary rates are higher for the M lineages than for the corresponding F lineages (Fig. 3)*.*

The presented arguments show that the two divergent mitogenomes of *S. algosus* are part of the DUI system. Consequently, it can be concluded that hermaphroditic bivalves can have the DUI system and that this system is associated with gonad development, rather than sex determination. The number of M-mtDNA copies present in a single sperm cell of *S. algosus*, was estimated at approximately 3 (Fig. 6). This number is in good agreement with the estimates of 1 up to 10 reported in mammals^45,55,56^. Our estimate may not be very precise, but the error should not exceed one copy per mitochondrion. The overall linear relationship between the M- and F- mtDNA concentration in the male mantle is intriguing. In DUI animals, the fate of paternal mtDNA in zygotes has been tracked through the first stages of development^18,57–59^. At these early stages, the advantage of F-type mtDNA is overwhelming in all cells, even those containing M-type mtDNA. Despite that, there is no F-type mtDNA in sperm, only M-type is present there. It has been suggested previously, that this elimination is a competitive process, possibly mediated by replicative advantage of M-type mtDNA over F-type mtDNA^60–62^. Our results suggest that the dominance of M mitogenome in sperm is achieved by a mild stepwise process and does not involve sudden expansion of the M-mitogenome. Further studies, involving other tissues and developmental stages should clarify this. *S. algosus* appears to be a perfect model system for DUI research as each individual has both mitogenomes, essentially eliminating the sex factor inherent to all previous DUI studies. These should ultimately allow identification of molecular pathways involved in mitochondria inheritance in DUI bivalves and a better understanding of mitochondrial inheritance in general.

## Methods

*Semimytilus algosus* specimens were collected in January 2014 at the Chilean Pacific coast (33°29′09.60″S, 71°38′40.97″W). The presence of male and female gonads was determined by mantle tissue examination under light microscopy. Sectioned gonad tissues were suspended in 70% Ethyl alcohol and stored in − 80 °C until further use. RNA and DNA was extracted according to the methods established earlier^63–65^. Tissue samples for DNA extraction (~ 60 mg) were incubated overnight in a 700 µl CTAB extraction buffer (2% CTAB, 0.1 M Tris-HCl, 1.4 M NaCl, 20 mM EDTA, 1 mg/ml proteinase K and 35 mM 2-mercaptoethanol) followed by triple chloroform extraction (1:1 vol/vol) and centrifugation (20,000×*g* for 10 min). Next, the aqueous phase was mixed with cold isopropanol (1:1 vol/vol) and centrifugated again (20,000×*g* for 30 min 4 °C). Retained DNA pellets were washed two times with 75% ethyl alcohol and dried in a vacuum concentrator (60 °C, 15 min). Isolated DNA were resuspended in a Tris-EDTA buffer and the concentrations were measured on a Epoch Microplate Spectrophotometer. RNA was extracted with GenElute Mammalian Total RNA Miniprep Kit (Sigma) optimized for mussel tissues, e.g. the original extraction buffer was exchanged for a CTAB extraction buffer. RNA Integrity was measured in a Epoch Microplate Spectrophotometer (A260/A280 ratio) and by gel electrophoresis.

Pooled total RNA extracts from three individuals (only male mantle) were then sent to Macrogen Inc. for high throughput sequencing (MiSeq Illumina, TruSeq NGS library 2 × 150 bp). Similarly, total DNA from single male mantle extract was sent for sequencing (HiSeq-X Ten Illumina, TruSeq NGS library 2 × 150 bp). Raw reads are available in the SRA GenBank database under the following accession numbers: SRR11805503*,* SRR11809905*.*

Raw reads after RNA sequencing were then processed and assembled according to the Oyster River protocol^66^ and the methodology published earlier^42,65,67^. ABySS^68^ was used as a nuclear genome assembler as well as NOVOplasty^69^, to pull out and assemble mitochondrial genomes from raw DNA reads. Sequences of assembled mtDNA were then verified by mapping of mitochondrial reads filtered with Bowtie2^70^ to the mitogenomes in CLC Genomics Workbench 9.5 (QIAGEN). Both acquired mitogenomes were then annotated following established workflow^42,71^. Proteins were predicted with CRITICA^72^, Wise2^73^ and GLIMMER^74^. Ribosomal genes (*tRNAs* and *rRNAs*) were identified with Infernal^75^, ARWEN^76^ and nhmmer^77^. The location of transmembrane protein domains was predicted with a Phobius^78^. A custom Biopython script was used to calculate compositional indices, synchronize the annotations and draw circular mitochondrial diagrams (https://github.com/aburzynski/mitoconstrictor). More details about the bioinformatic pipeline can be found in the supplementary materials. The annotated features were then verified manually in CLC Genomics Workbench 9.5. Annotated mitochondrial sequences of *Semimytilus algosus* are available in the GenBank database under accession numbers MT026712 and MT026713.

To identify the allelic coverage of the nuclear genome, Jellyfish^79^ analyses were performed. K-mer coverage values between the homozygous and heterozygous peaks (2N-1N alleles) were set for target single copy nuclear gene identification (Fig. S3). This was performed in order to exclude genes with more than two alleles which would indicate the existence of more than one gene copy. Nuclear genes were chosen by non-random method: pick a known gene annotated in *Crassostrea gigas* (GCA_000297895.1) genome^80,81^, search it with BLAST^82^ and Wise2^73^ for its counterpart in *Semimytilus algosus* transcriptome, then map identified transcripts onto the *S. algosus* assembled genome*.* Filter out mapped reads with Bowtie2^70^ and check the k-mer coverage (repeat until a suitable gene is identified). Two pairs of nuclear primers and six pairs of mitochondrial (3 pairs for F and 3 pairs for M mtDNA) primers were designed in Primer3^83^ for a qPCR reaction. As a result of primer optimisation (Efficiency between 90 and 110%) only three pairs of primers remained (Nuclear: *ATP asynthase alpha subunit*, F mtDNA: *cox1*, M mtDNA: *nad1*). Primers for the F and M-type mitogenomes were experimentally checked to ensure no amplification of homologous sequences from the second mitogenome. For every gene a freshly diluted standard curve (PCR amplified template spanning gene of interest) was run along the samples and a non template control. The final standard curve was established as an average for three experiments (3 experiments × 3 replicates × 7 folds of dilution).

QPCR reactions verifying amounts of F and M mtDNA in tissues were performed on a ECO48 (Illumina) Real Time PCR System according to protocol supplied by the manufacturer (EURx): a reaction volume of 10 µl per sample, 1 × SG qPCR Master Mix, 2 µl of DNA concentration ~ 11 ng/µl linearized with a NdeI restriction enzyme, 0.5 µM of primers. Thermal profile: initial denaturation 95 °C for 10 min followed by 35 cycles of denaturation 10 s 94 °C, annealing 60 °C 30 s, elongation 72 °C 30 s, melt curve 55–95 °C. Primers sequences and raw results are available in Table S4–S7.

Phylogenetic reconstruction of the Mytilidae family^67,84^ were performed in BEAST2^85^ on 12 mitochondrial protein sequences (Fig. 3). Complete mitochondrial genomes of 41 individuals (8 F/M pairs and 32 species) were downloaded from the GenBank database in November 2019 (Table 2). MEGA7^86^ has been used for sequence alignment and p-distance divergence calculations. Protein sequences were aligned using ClustalW algorithm with Gap Extension and Gap Opening cost set at 5. Parameters for phylogenetic analysis were as follows: mtREV model, relaxed log-normal clock and Yule prior to the common tree. Markov chain was run in four replicates for 10^7^ generations and sampled every 10,000^th^ step. Convergence of samples was checked with Tracer^87^, effective sample size for estimated parameters was greater than 200.

**Table 2:** List of mitogenomes used in phylogenetic analyses, with species, lineage, accession numbers, and references (wherever available).

Species and references	Acc. No	Species and references	Acc. no
*Bathymodiolus japonicus*^88^	AP014560	*Mytilus californianus* F^89^	GQ527172
*Bathymodiolus platifrons*^88^	AP014561	*Mytilus californianus* M^89^	GQ527173
*Bathymodiolus septemdierum*^88^	AP014562	*Mytilus chilensis*^90^	KT966847
*Bathymodiolus thermophilus*^84^	MK721544	*Mytilus coruscus*^91^	KJ577549
*Brachidontes exustus*^92^	KM233636	*Mytilus edulis* F^38^	MF407676
*Brachidontes mutabilis*^84^	MK721541	*Mytilus edulis* M^93^	HM489874
*Crenomytilus grayanus*^84^	MK721543	*Mytilus galloprovincialis* F^94^	FJ890850
*Geukensia demissa* F^67^	MN449487	*Mytilus galloprovincialis* M^94^	FJ890849
*Geukensia demissa* M^67^	MN449488	*Mytilus trossulus* F^39^	HM462080
*Gregeriella coralliophaga*^84^	MK721545	*Mytilus trossulus* M^39^	HM462081
*Limnoperna fortunei*^95^	KP756905	*Perna canaliculus*^96^	MG766134
*Lithophaga curta*^84^	MK721546	*Perna perna*	(unpublished)
*Modiolus kurilensis*	KY242717	*Perna viridis*^97^	JQ970425
*Modiolus modiolus*^98^	KX821782	*Perumytilus purpuratus* NF^42^	MH330322
*Modiolus nipponicus*^84^	MK721547	*Perumytilus purpuratus* NM^42^	MH330330
*Modiolus philippinarum*^99^	KY705073	*Perumytilus purpuratus* SF^42^	MH330333
*Musculista senhousia* F^41^	GU001953	*Perumytilus purpuratus* SM^42^	MH330331
*Musculista senhousia* M^41^	GU001954	*Semimytilus algosus* F	MT026712
*Mytilaster solisianus*^100^	KM655841	*Semimytilus algosus* M	MT026713
*Mytilisepta keenae*^84^	MK721542	*Septifer bilocularis*^84^	MK721549
*Mytilisepta virgata*^84^	MK721548		

Two sets of primers covering fragments of the three genes have been designed^83^ to evaluate selective pressure (Table 1) acting on F and M-type mtDNA. DNA from 18 pairs of female and male mantle tissue samples was extracted using a modified CTAB protocol^63^. PCR amplifications were carried out in a 12 μl reaction volume with approximately 5 ng of total DNA, 0.5 μM each primer, dNTPs at 200 μM each, 0.02 U/μl of Phusion DNA Polymerase (Thermo Fisher Scientific) in a GC buffer supplied by the manufacturer. The TProfessional Thermocycler (Biometra) was used to perform amplification reactions according to the following protocol: 30 s at 98 ˚C initial denaturation, 30 cycles of denaturation for 10 s at 98 ˚C, annealing for 30 s at temperature dependent on primers (Table S8) and extension for 45 s at 72 ˚C. The final extension lasted 10 min. Amplified products where then sent for sequencing to Macrogen Inc. (Korea). The Gap4 software from the Staden package version 1.7.0^101^ was used to assemble sequences. Alignments and sequence analysis were performed using MEGA7^86^ and DnaSP^102^ software. The nucleotide divergences of protein genes in synonymous (Ks) and non-synonymous sites (Ka) using the Nei–Gojobori method with Jukes–Cantor correction were calculated in MEGA7. The calculations of the D test statistic proposed by Tajima^103^ was performed in DnaSP^102^.

## Supplementary information

Supplementary information
