# Supplementary material for: Semimytilus algosus: first known hermaphroditic mussel with doubly uniparental inheritance of mitochondrial DNA
Source: Sci Rep. 2020 Jul 9;10:11256. doi: 10.1038/s41598-020-67976-6 (PMC7347871; doi:10.1038/s41598-020-67976-6)
Supplement: Supplementary file 1 — Supplementary information [file 41598_2020_67976_MOESM1_ESM.pdf]

Supplementary Information for:

Semimytilus algosus - first known hermaphroditic mussel with doubly uniparental inheritance of mitochondrial DNA

Marek Lubośny[1], Aleksandra Przyłucka[1], Beata Śmietanka[1], Artur Burzyński[1]

[1] Department of Genetics and Marine Biotechnology, Institute of Oceanology Polish Academy of Sciences, Sopot, Poland

[*]Corresponding Author: Marek Lubośny (lubosny@iopan.pl)

This PDF file includes:

Figures S1 to S3 (Page 7-9)
Tables S1 to S8 (Page 2-6, 10)
Methodology S1 (Page 10-11)

Table S1. Average coverage of F and M *Semimytilus algosus* mitogenome with NGS data.

	coverage RNAseq		coverage DNAseq	
Sequence	F RNA	M RNA	F DNA	M DNA
cytb	4867.25	35.94	1461.02	44.16
cox1	8654.00	19.96	1588.69	49.99
cox2	6310.67	27.18	1545.02	50.36
cox3	6741.89	78.60	1657.24	45.47
atp6	6020.49	19.17	1652.69	60.99
atp8	266.79	8.81	1743.98	51.89
nad1	3110.11	47.67	1602.79	54.99
nad2	2467.80	62.86	1645.11	49.29
nad3	2132.50	20.56	1552.68	53.63
nad4	1434.67	20.47	1389.74	53.66
nad4l	1456.33	18.61	1667.92	47.58
nad5	2111.67	8.85	1593.75	50.01
nad6	2477.73	43.19	1388.54	41.00
16S rRNA	79474.15	250.97	1601.05	48.08
12S rRNA	2750.65	40.65	1620.47	50.77
Whole mtDNA	8183.15	38.02	1603.20	51.63

Extended Data Table S2. qPCR Standard curves efficiency

Gene	Equation	Efficiency	R^2
ATPα Nuclear	y=-3.43x+35.03	95.58%	0.999
Cox1 F mtDNA	y=-3.5x+35.39	92.93%	0.994
Nad1 M mtDNA	y=-3.57x+35.623	90.61%	0.996

Extended Data Table S3. Sequences of the qPCR primers

Gene	5’-Sequence -3’
N_ATPα Frw	TGTAGCTTACCGTCAGATGTCTCTG
N_ATPα Rev	GTTTCAATGACTGGCAATGCTG
F_Cox1 Frw	GCAGTTGATCTGGCAATTTTGTC
F_Cox1 Rev	AACATATAAGACGGAACGCTCTCC
M_Nad1 Frw	GGTTTTTCACTCTGTTGGAGCG
M_Nad1 Rev	TCCCTAAAAGGCTGAAGAAGACC

Table S4. qPCR results for nuclear gene *atpα*

Sample ATPα_N	ng/sample	Cq	Cq SD	Copies	Copies + SD	Copies - SD
PL01F	9.54	24.61	0.13	1091.19	1190.70	1000.00
PL01M	27.10	21.15	0.06	11133.90	11595.81	10690.38
PL02F	30.85	23.23	0.07	2755.71	2888.29	2629.21
PL02M	23.61	24.4	0.01	1256.39	1264.86	1247.99
PL03F	35.12	25.93	0.11	449.84	484.32	417.82
PL03M	20.07	22.22	0.00	5428.68	5428.68	5428.68
PL04F	16.14	24.45	0.06	1214.92	1264.86	1166.96
PL04M	24.58	23.1	0.08	3007.00	3172.91	2849.77
PL05F	31.78	26.09	0.02	404.03	409.49	398.64
PL05M	25.49	22.3	0.05	5144.82	5320.44	4975.00
PL11F	24.12	24.2	0.16	1436.93	1599.86	1290.59
PL11M	23.87	21.42	0.14	9288.17	10203.43	8455.00
PL13F	25.81	24.06	0.03	1578.52	1610.63	1547.05
PL13M	29.76	20.77	0.07	14369.26	15060.61	13709.65
PL14F	22.99	23.46	0.11	2361.45	2542.42	2193.35
PL14M	22.97	21.12	0.11	11360.40	12231.04	10551.73
PL15F	29.33	24.35	0.07	1299.28	1361.79	1239.64
PL15M	33.33	20.33	0.05	19306.98	19966.02	18669.69
CH04F	19.40	24.24	0.12	1398.85	1516.21	1290.59
CH04M	29.50	19.04	0.03	45899.46	46833.21	44984.33
CH07F	21.80	23.69	0.04	2023.59	2078.66	1969.97
CH07M	16.70	25.77	0.03	500.85	511.04	490.87
CH12F	26.60	23.32	0.06	2594.14	2700.77	2491.73
CH12M	18.65	21.52	0.29	8685.11	10551.73	7148.71
CH33F	21.50	23.61	0.07	2135.24	2237.97	2037.22
CH33M	25.60	19.57	0.02	32157.97	32592.64	31729.10
CH08F	17.51	21.67	0.03	7853.15	8012.91	7696.57
CH08M	22.99	20.2	0.02	21067.60	21352.37	20786.64
PL17F	29.71	24.14	0.02	1495.98	1516.21	1476.03
PL17M	17.34	24.6	0.09	1098.54	1166.96	1034.14
PL19F	21.12	23.74	0.06	1956.79	2037.22	1879.54
PL19M	16.64	20.83	0.04	13801.99	14177.63	13436.31
PL20F	20.28	25.38	0.04	650.75	668.46	633.50
PL20M	21.06	24.62	0.23	1083.89	1264.86	928.82
NTC	-	32.54	1.45	-	-	-

Cq - quantitation cycle; SD – standard deviation; NTC – no template control; F – female gonad/mantle; M – male gonad/mantle; PL00/CH00 – interior sample coding number;

Table S5. qPCR results for F-type mitochondrial gene *cox1*

Sample Cox1_F	ng/sample	Cq	Cq SD	Copies	Copies + SD	Copies - SD
PL01F	9.54	15.84	0.08	385224.84	406042.44	365474.55
PL01M	27.10	19.66	0.08	31312.25	32944.59	29760.79
PL02F	30.85	15.43	0.08	504495.32	531758.31	478630.09
PL02M	23.61	17.21	0.02	156417.63	158489.32	154373.03
PL03F	35.12	16.21	0.02	301995.17	305994.97	298047.66
PL03M	20.07	20.73	0.03	15437.30	15745.01	15135.61
PL04F	16.14	14.59	0.02	876712.39	888324.07	865252.49
PL04M	24.58	16.33	0.11	279070.73	300014.92	259588.66
PL05F	31.78	15.75	0.01	408722.52	411420.30	406042.44
PL05M	25.49	18.77	0.06	56049.46	58306.14	53880.12
PL11F	24.12	14.54	0.03	906030.58	924090.03	888324.07
PL11M	23.87	17.09	0.01	169266.66	170383.91	168156.74
PL13F	25.81	14.93	0.05	700993.98	724435.96	678310.55
PL13M	29.76	16.60	0.12	233653.04	252846.61	215916.44
PL14F	22.99	14.60	0.09	870963.59	924090.03	820891.42
PL14M	22.97	18.15	0.09	84278.01	89418.75	79432.82
PL15F	29.33	13.82	0.06	1454980.40	1513561.25	1398666.86
PL15M	33.33	16.67	0.20	223136.92	254515.53	195626.90
CH04F	19.40	13.70	0.10	1574500.70	1681567.43	1474250.99
CH04M	29.50	18.42	0.10	70562.09	75360.34	66069.34
CH07F	21.80	15.32	0.05	542357.58	560494.59	524807.46
CH07M	16.70	19.68	0.05	30801.47	31831.50	29804.77
CH12F	26.60	13.02	0.02	2462796.75	2495415.44	2430604.43
CH12M	18.65	20.82	0.14	14549.80	15953.54	13269.58
CH33F	21.50	13.60	0.15	1681567.43	1855972.04	1523551.51
CH33M	25.60	18.96	0.13	49463.60	53880.12	45409.10
CH08F	17.51	15.12	0.01	618626.57	622709.82	614570.10
CH08M	22.99	18.12	0.02	85957.88	87096.36	84834.29
PL17F	29.71	14.23	0.01	1111000.58	1118333.74	1103715.50
PL17M	17.34	18.18	0.09	82630.97	87671.24	77880.47
PL19F	21.12	17.11	0.03	167054.10	170383.91	163789.37
PL19M	16.64	17.89	0.08	100000.00	105404.01	94873.05
PL20F	20.28	17.04	0.02	174927.12	177243.95	172640.57
PL20M	21.06	18.91	0.15	51117.71	56419.41	46314.21
NTC	-	32.66	1.15	-	-	-

Cq - quantitation cycle; SD – standard deviation; NTC – no template control; F – female gonad/mantle; M – male gonad/mantle; PL00/CH00 – interior sample coding number;

Table S6. qPCR results for M-type mitochondrial gene *nad1*

Sample Nad1_M	ng/sample	Cq	Cq SD	Copies	Copies + SD	Copies - SD
PL01F	9.54	25.04	0.03	919.571	937.538	901.949
PL01M	27.10	16.75	0.18	193070	217905	171065
PL02F	30.85	27.64	0.12	171.907	185.741	159.104
PL02M	23.61	22.22	0.11	5668.93	6085.74	5280.67
PL03F	35.12	>31.32	NON	<16	SPECYFIC	AMPLIF.
PL03M	20.07	17.29	0.1	136287	145367	127774
PL04F	16.14	22.02	0.07	6449.47	6747.33	6164.76
PL04M	24.58	17.67	0.15	106662	117497	96826.5
PL05F	31.78	>31.29	NON	<16	SPECYFIC	AMPLIF.
PL05M	25.49	16.76	0.04	191829	196842	186943
PL11F	24.12	21.79	0.11	7480.83	8030.86	6968.47
PL11M	23.87	15.32	0.07	485595	508022	464159
PL13F	25.81	21.23	0.11	10735.3	11524.6	10000
PL13M	29.76	14.74	0.12	705894	762699	653320
PL14F	22.99	26.3	0.06	407.985	424.083	392.498
PL14M	22.97	15.92	0.14	329767	360930	301295
PL15F	29.33	20.87	0.16	13541.1	15013.1	12213.3
PL15M	33.33	14.43	0.05	862133	890389	834773
CH04F	19.40	>29.80	NON	<42	SPECYFIC	AMPLIF.
CH04M	29.50	14.55	0.08	797923	840175	757795
CH07F	21.80	>29.76	NON	<43	SPECYFIC	AMPLIF.
CH07M	16.70	21.04	0.05	12134.8	12532.5	11749.7
CH12F	26.60	22.13	0.07	6007.74	6285.2	5742.53
CH12M	18.65	17.54	0.12	115991	125325	107353
CH33F	21.50	25.63	0.04	628.52	644.947	612.512
CH33M	25.60	15	0.04	596912	612512	581709
CH08F	17.51	18.36	0.06	68349.3	71046.2	65754.8
CH08M	22.99	15.1	0.1	559628	596912	524672
PL17F	29.71	23.26	0.01	2898.58	2917.33	2879.94
PL17M	17.34	22.64	0.07	4323.69	4523.37	4132.82
PL19F	21.12	25.11	0.02	878.977	890.389	867.711
PL19M	16.64	15.21	0.06	521299	541868	501511
PL20F	20.28	27.01	0.24	258.086	301.295	221.074
PL20M	21.06	18.45	0.11	64494.7	69236.7	60077.4
NTC	-	32.69	1.71	-	-	-

Cq - quantitation cycle; SD – standard deviation; NTC – no template control; F – female gonad/mantle; M – male gonad/mantle; PL00/CH00 – interior sample coding number;

Table S7. mtDNA to nDNA ratio for F and M mitochondrial genome

Sample	cox1/atpα	SD +	SD -	Sample	nad1/atpα	SD +	SD -
PL01F	353.03	53.01	46.09	PL01F	0.84	0.09	0.09
PL01M	2.81	0.27	0.25	PL01M	17.34	3.04	2.59
PL02F	183.07	19.18	17.36	PL02F	0.06	0.01	0.01
PL02M	124.50	2.50	2.45	PL02M	4.51	0.36	0.34
PL03F	671.33	61.02	55.94	PL03F	0.04	-	-
PL03M	2.84	0.06	0.06	PL03M	25.10	1.67	1.57
PL04F	721.62	39.61	37.55	PL04F	5.31	0.47	0.43
PL04M	92.81	12.47	10.99	PL04M	35.47	5.76	4.95
PL05F	1011.61	20.44	20.04	PL05F	0.04	-	-
PL05M	10.89	0.83	0.77	PL05M	37.29	2.28	2.15
PL11F	630.53	85.49	75.28	PL11F	5.21	1.02	0.85
PL11M	18.22	1.93	1.74	PL11M	52.28	7.80	6.79
PL13F	444.08	24.19	22.94	PL13F	6.80	0.65	0.59
PL13M	16.26	2.18	1.92	PL13M	49.13	6.51	5.75
PL14F	368.83	52.49	45.95	PL14F	0.17	0.02	0.02
PL14M	7.42	1.06	0.92	PL14M	29.03	5.18	4.39
PL15F	1119.84	101.14	92.76	PL15F	10.42	1.69	1.45
PL15M	11.56	2.08	1.76	PL15M	44.65	3.04	2.84
CH04F	1125.56	177.38	153.23	CH04F	0.03	-	-
CH04M	1.54	0.14	0.13	CH04M	17.38	1.29	1.20
CH07F	268.02	16.50	15.54	CH07F	0.02	-	-
CH07M	61.50	3.35	3.18	CH07M	24.23	1.30	1.24
CH12F	949.37	52.11	49.40	CH12F	2.32	0.21	0.19
CH12M	1.68	0.56	0.42	CH12M	13.36	4.18	3.18
CH33F	787.53	123.50	106.76	CH33F	0.29	0.02	0.02
CH33M	1.54	0.16	0.14	CH33M	18.56	0.74	0.71
CH08F	78.77	2.13	2.08	CH08F	8.70	0.53	0.50
CH08M	4.08	0.11	0.11	CH08M	26.56	2.15	1.99
PL17F	742.66	15.01	14.71	PL17F	1.94	0.04	0.04
PL17M	75.22	9.56	8.48	PL17M	3.94	0.44	0.39
PL19F	85.37	5.28	4.97	PL19F	0.45	0.02	0.02
PL19M	7.25	0.60	0.55	PL19M	37.77	2.56	2.40
PL20F	268.81	10.97	10.54	PL20F	0.40	0.08	0.07
PL20M	47.16	13.58	10.55	PL20M	59.50	15.04	12.01
Avg. F	577.06	340.79	340.79	Avg. F	2.53	3.32	3.32
Avg. M	28.66	36.63	36.63	Avg. M	29.18	15.73	15.73

SD – standard deviation; Avg – average value; F – female gonad/mantle; M – male gonad/mantle; PL00/CH00 – interior sample coding number; cox1/atpα – represents copies of F-type mtDNA divided by nuclear DNA copy number; nad1/atpα - represents copies of M-type mtDNA divided by nuclear DNA copy number

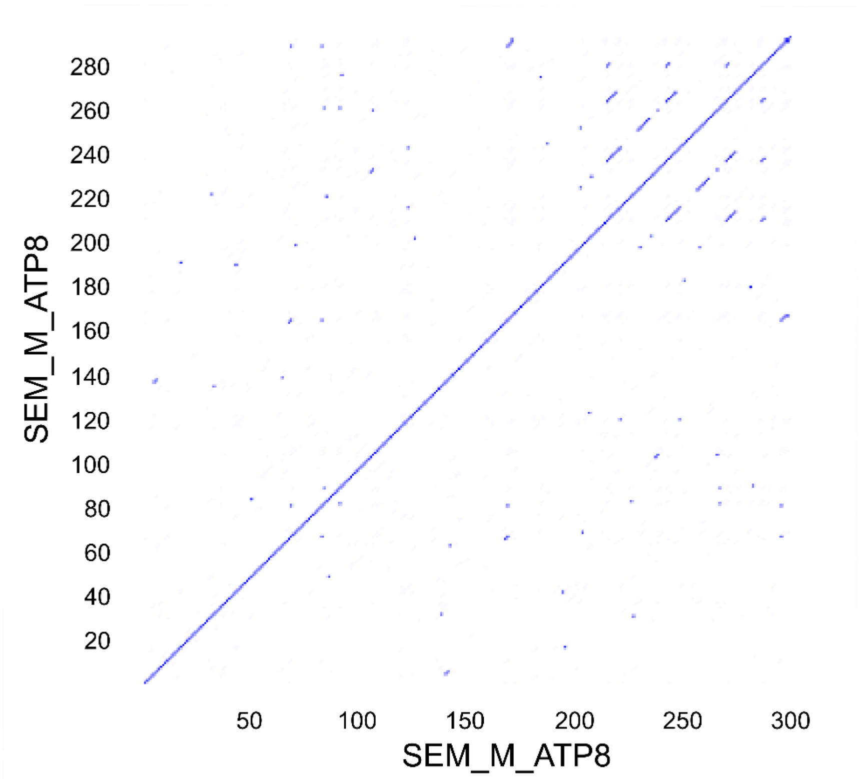

Figure S1.Dot plot for ATP8 protein sequence from M-mtDNA of *Semimytilus algosus*. Amino acid sequence of ATP8 gene from M -type mitogenome was plotted against itself in CLC Genomic workbench 9.5 with widow size parameter set to 3. Presence of few 7-9 amino acid longs repeats has been checked by manual examination of protein sequence in MEGA7.

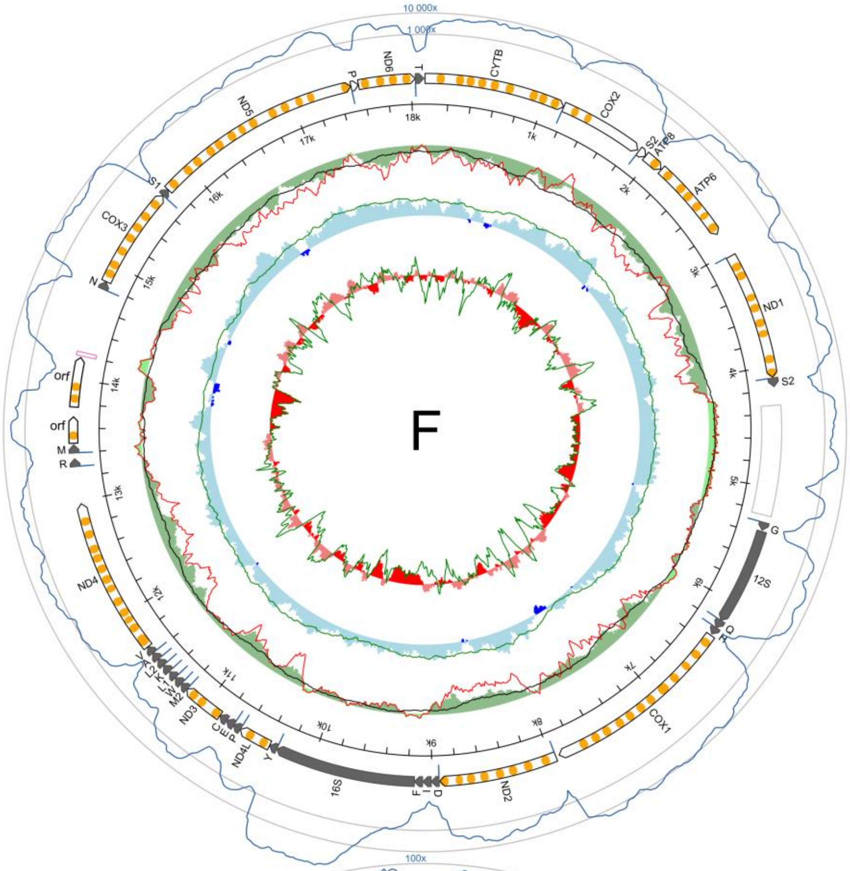
10 000x
1 000x
F
CYTB
COX2
ATP8
ATP6
ND1
S2
G
12S
Q
COX1
ND2
16S
ND4L
ND3
M2
ND4
orf
orf
M
R
N
COX3
S1
ND5
P
ND6
T
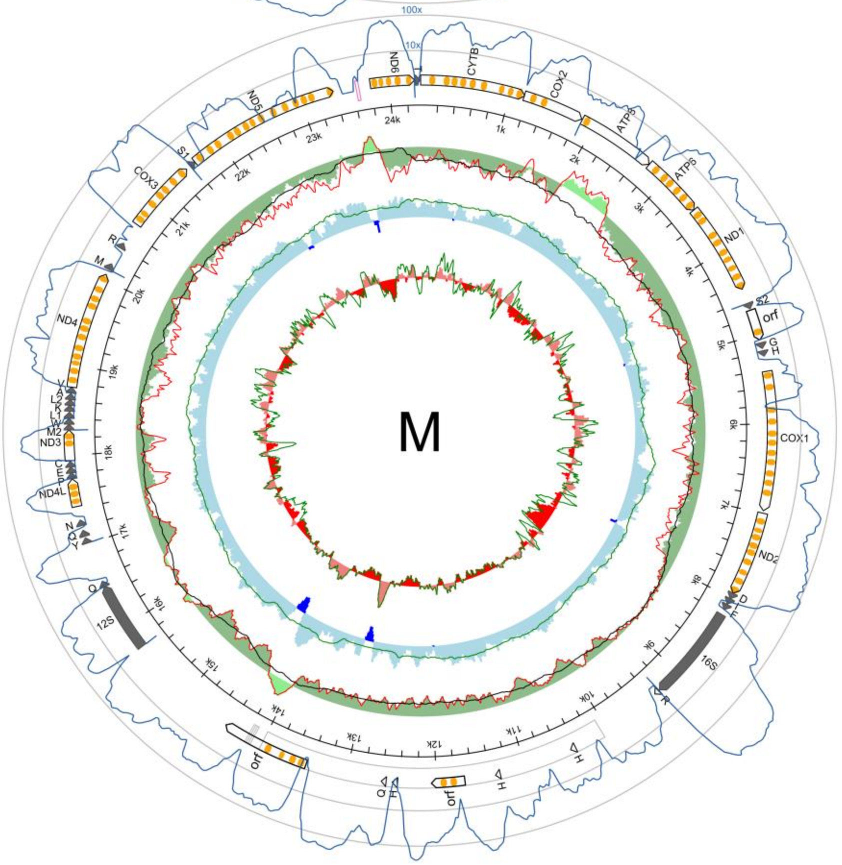
100x
10x
M
ND6
CYTB
COX2
ATP8
ATP6
ND1
S2
orf
G
H
COX1
ND2
D
16S
R
orf
orf
12S
Q
N
ND4L
ND3
M2
ND4
M
R
COX3
S1
ND5

Figure S2. Genetic map of *Semimytilus algosus* mitochondrial genomes. The white arrows represent protein coding genes with predicted transmembrane domains of encoded proteins marked in orange. The long dark arrows represent rRNA genes; the short dark arrows represent tRNA labeled by a one letter amino acid code, short white arrows represent duplicated tRNA-like structures, a pink box indicates the location of an AT-rich region; a grey box indicates the location of repetitive sequences; blue lines in front of the genes show the location of the polyadenylation signal. Inner circles represent local compositional bias, calculated in a 200 bp long sliding window with 25 bp steps, unless indicated otherwise. A green outer circle represents a AT-skew (A−T/A+T); a red line represents a filtered AT-skew, calculated at non-coding regions and the second codon position only. A black line represents a filtered AT-skew, calculated at neutral and non-coding positions only, in a larger, 1000bp long window. A middle blue circle represents a GC skew (G−C/G+C), and a green line represents a GC skew at neutral sites, calculated in a window of 1,000 bp. Both skew indices are presented in absolute scale, starting at zero. An inner red circle represents local GC content, and a green line shows GC content at neutral sites. Local GC content is presented in scale relative to the average for the whole mitogenome. Grey circles represent coverage: 1000× and 10000× for F mitogenome and 10× and 100× for M mitogenome.

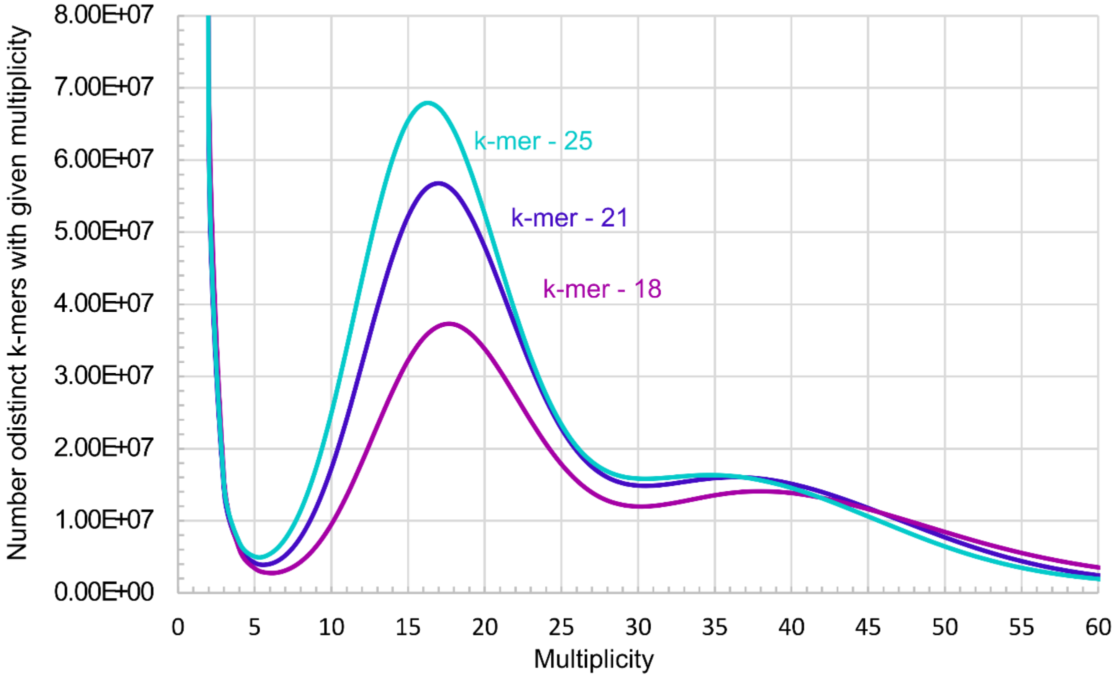

Figure S3. Jellyfish analysis for NGS DNAseq data. Analysis shows two peaks first peak shows kmer coverage for heterozygotic locations in genome, second peak shows kmer coverage for homozygotic locations in genomes.

Table S8. Sequences of the PCR primers for population studies

Primer	mtDNA	5’-Sequence -3’	Temp
SEM_G<br>SEM_O	F	AGATGGTCAGCCTTATTTGTATAG<br>CTCAGTACCAGGAATATCCCTAC	61.3 ºC
SEM_G_male<br>SEM_O_male	M	CAGGATGGCAACGCTTATC<br>AACCTAACACCAAAAACATAACAAT	66 ºC
SEM71R<br>SEM20R	F	TGGCTAAAGTTGTAGATTAGGCTG<br>CCATGTCCATAGAATTTAGGAGTC	61.3 ºC
SEM2110F<br>20R_male	M	AGCTTCTGTGTGGTATTGGC<br>AGATTCCTAGCCGTAACATACAA	69.4 ºC

Methodology S1: Bioinformatic pipeline details

Raw RNA-seq reads were processed according to Oyster River protocol[60]: they were trimmed and filtered against potential contaminating adaptor sequences first, then assembled. CLC Genomics Workbench 9.5 (QIAGEN) was used for that, according to the manual available from the manufacturer.

This RNA-seq assembly was used to identify potential mitotranscripts. To this end protein profiles (hmms) corresponding to mitochondrially encoded OXPHOS components were downloaded from Pfam database. Then, these profiles were used to search the obtained transcriptome assembly with wise2[67], using the following command line options:

genewise <profile> <transcriptome> -codon table5 -hmmer

where <profile> and <transcriptome> indicate file names of the downloaded hmm profiles and RNA-seq assembly, respectively. Manual inspection of the results allowed identification of two sets of contigs for cox1, cox2, cox3, atp6 and cytb. These sequences were used as baits in subsequent steps.

Raw genomic reads from shotgun TrueSeq Illumina library were trimmed and assembled, under default settings, with ABySS[62]. This assembly was used primarily as a source of reference genomic sequences but the mitogenomic contigs were also identified in this assembly by identical methodology as for transcriptome assemblies. To obtain complete, circular assemblies of mitogenomes, dedicated assembler, NOVOplasty[63] was used. All the settings for this assembler were at the default values and bait sequences obtained in the previous step were used. After obtaining the complete circular assembly, its correctness was verified by mapping the raw sequencing reads back at the assembled mitogenomes. This was achieved by running bowtie2,

under the default parameters. Additionally, mapped reads were imported into CLC Genomics Workbench and the mapping was repeated there, followed my manual inspection of its quality.

Annotation of the obtained sequences followed the established protocol[41,65].

Proteins were predicted with CRITICA[66], Wise2[67] and GLIMMER[68]. Ribosomal genes (*tRNAs* and *rRNAs*) were identified with Infernal[69,70] and nhmmer[71]. Localization of transmembrane protein domains were predicted with Phobius[72]. All these programs were run under default parameters, except for obligatory changes involving file names, genetic code (NCBI table 5 was used) and using RefSeq mitogenomes as reference sequences when needed.
